# Supplementary material for: Complementary, alternative, and integrative medicine researchers’ practices and perceived barriers related to open science: An international, cross-sectional survey
Source: PLoS One. 2024 May 6;19(5):e0301251. doi: 10.1371/journal.pone.0301251 (PMC11073706; doi:10.1371/journal.pone.0301251)
Supplement: S1 File — (DOCX) [file pone.0301251.s001.docx]

**Checklist for Reporting Results of Internet E-Surveys (CHERRIES)**

| ***Item category*** | ***Checklist Item*** | ***Explanation*** | ***Page Number*** |
| --- | --- | --- | --- |
| Design | Describe survey design | The study involved a convenience sample. Journals containing the words “complementary”, “alternative” or “integrative” in their names were chosen from Table 2 of Ng (citation #27 in the manuscript). This list contains journals belonging to the Scopus category “complementary and alternative medicine” (code 2707) which were identified based on the All Science Journal Classification. We searched for all articles published in each journal using the ISSN number of included journals. We ran a search for each journal separately. After each search, we sorted the results by Entry date (descending) and export all publications from January 1, 2018 to December 31, 2022. The list of PMID numbers were exported as an .csv file and inputted into an R script (built based on the easyPubMed package) to retrieve the authors’ name, affiliation institutions and email addresses. | Pg 5-7, Table 2 |
| Ethics | IRB approval | This study was approved by the Ottawa Health Sciences Research Ethics Board. | Pg 5 |
|  | Informed consent | Upon clicking the survey link in the invitation email, participants had to read and agree with the informed consent form before being able to see the survey. It did not require a signature due to the anonymous nature of the survey. In the informed consent form, participants were told the purpose of the study, the length of time to complete the survey, principal investigator name, and data storage information. Please see the Supplementary files for the informed consent form and survey. | Pg 5-7 |
|  | Data protection | No personally identifying information was collected | Pg 5-7 |
| Development and pre-testing | Development and testing | The survey was created based on previous literature, and pilot-tested prior to being released. | Pg 5-7 |
| Recruitment process | Open survey versus closed survey | The data was collected using a closed survey. | Pg 5-7 |
|  | Contact mode | Initial contact with participants was made on the Internet. An email was sent to participants with a link, inviting them to complete the survey. | Pg 5-7 |
|  | Advertising the survey | The survey was not announced or advertised. Randomly selected authors were invited to participate. | Pg 5-7 |
| Survey administration | Web/E-mail | The survey was sent to participants via email using SurveyMonkey. Data was entered automatically when participants responded to the questions, and collected through SurveyMonkey. | Pg 5-7 |
|  | Context | SurveyMonkey is a cloud-based survey tool that allows you to easily create surveys that can be sent to participants through weblinks, emails or embedding to websites. It collects participants’ responses and provides analysis on the website, or exportation to other software. | None |
|  | Mandatory/voluntary | This was a voluntary survey. | 5-7 |
|  | Incentives | No incentives were offered. | 5-7 |
|  | Time/Date | Responses were collected from February-March 2023. | Pg 6 |
|  | Randomization of items or questionnaires | No randomization of items was used. | None |
|  | Adaptive questioning | Adaptive questioning was used. | 5-7 |
|  | Number of Items | The survey contained 34 questions. | 5-7 |
|  | Number of screens (pages) | The survey was distributed over 12 pages. | 5-7 |
|  | Completeness check | Completeness was checked manually after the survey was submitted | 5-7 |
|  | Review step | Participants could use a Back button to change their answers. However, once the completed survey was submitted to the study team, it was not possible to withdraw responses. | 5-7 |
| Response rates | Unique site visitor | We did not determine if responses were by a unique visitor. | None |
|  | View rate | We did not determine view rate. | None |
|  | Participation rate | We did not determine participation rate. | None |
|  | Completion rate | The survey had a completion rate of 95.48%. Incomplete responses were defined as responses with no questions answered after the second page of the survey. | Pg 8 |
| Preventing multiple entries from the same individual | Cookies used | Cookies were not used to identify potential duplicate entries from the same user. | None |
|  | IP check | IP addresses were not used to identify potential duplicate entries from the same user. | None |
|  | Log file analysis | No log file analysis was done. | None |
|  | Registration | No registration required, as invites were only given to randomly selected authors, and was not shared to others. | Pg 5-7 |
| Analysis | Handling of incomplete questionnaires | Questionnaires which terminated early were also analyzed. | Pg. 8 |
|  | Questionnaires submitted with an atypical timestamp | No respondents were removed from the survey for completing the items too quickly. | None |
|  | Statistical correction | No methods have been used to adjust for the non-representative sample. | None |

Eysenbach G. Improving the quality of Web surveys: the Checklist for Reporting Results of Internet E-Surveys (CHERRIES). J Med Internet Res. 2004 Sep 29;6(3):e34 [erratum in J Med Internet Res. 2012; 14(1): e8.]. Article available at [https://www.jmir.org/2004/3/e34](https://www.jmir.org/2004/3/e34/)/; erratum available <https://www.jmir.org/2012/1/e8/>.
