# Supplementary material for: Complementary, alternative, and integrative medicine researchers’ practices and perceived barriers related to open science: An international, cross-sectional survey
Source: PLoS One. 2024 May 6;19(5):e0301251. doi: 10.1371/journal.pone.0301251 (PMC11073706; doi:10.1371/journal.pone.0301251)
Supplement: Attachment — Submitted filename: Response to Reviewers_Feb0224.docx [file pone.0301251.s002.docx]

**Response to Reviewers**

Dear Editor:

Thank you for your email. I am pleased to hear that you believe that our manuscript will have the potential to be published in PLOS ONE following requested revisions.

As per your request, please find our response to the peer-reviewers’ comments with every change outlined point by point below:

=====================================================================

Journal Requirements:

- We kindly thank the editor for providing their feedback on our manuscript.

When submitting your revision, we need you to address these additional requirements.

1. Please ensure that your manuscript meets PLOS ONE's style requirements, including those for file naming. The PLOS ONE style templates can be found at

https://journals.plos.org/plosone/s/file?id=wjVg/PLOSOne_formatting_sample_main_body.pdf and

https://journals.plos.org/plosone/s/file?id=ba62/PLOSOne_formatting_sample_title_authors_affiliations.pdf

- We have ensured the manuscript meets PLOS ONE’s style requirements.

2. Please provide additional details regarding participant consent. In the ethics statement in the Methods and online submission information, please ensure that you have specified what type you obtained (for instance, written or verbal, and if verbal, how it was documented and witnessed). If your study included minors, state whether you obtained consent from parents or guardians. If the need for consent was waived by the ethics committee, please include this information.

- We have moved the ethics statement to the Methods which includes all consent information,

3. PLOS requires an ORCID iD for the corresponding author in Editorial Manager on papers submitted after December 6th, 2016. Please ensure that you have an ORCID iD and that it is validated in Editorial Manager. To do this, go to ‘Update my Information’ (in the upper left-hand corner of the main menu), and click on the Fetch/Validate link next to the ORCID field. This will take you to the ORCID site and allow you to create a new iD or authenticate a pre-existing iD in Editorial Manager. Please see the following video for instructions on linking an ORCID iD to your Editorial Manager account: https://www.youtube.com/watch?v=_xcclfuvtxQ

- We have ensured the ORCID iD is validated in the editorial manager.

4. Your ethics statement should only appear in the Methods section of your manuscript. If your ethics statement is written in any section besides the Methods, please move it to the Methods section and delete it from any other section. Please ensure that your ethics statement is included in your manuscript, as the ethics statement entered into the online submission form will not be published alongside your manuscript.

- We have moved the ethics statement to the methods section.

5. Please include captions for your Supporting Information files at the end of your manuscript, and update any in-text citations to match accordingly. Please see our Supporting Information guidelines for more information: http://journals.plos.org/plosone/s/supporting-information.

- We have included captions for the supporting information files.

6. Please review your reference list to ensure that it is complete and correct. If you have cited papers that have been retracted, please include the rationale for doing so in the manuscript text, or remove these references and replace them with relevant current references. Any changes to the reference list should be mentioned in the rebuttal letter that accompanies your revised manuscript. If you need to cite a retracted article, indicate the article’s retracted status in the References list and also include a citation and full reference for the retraction notice.

- We have ensured the reference list is complete and correct.

Additional Editor Comments:

Please make sure that the manuscript is consistent throughout the text. As the reviewers indicate, this is particularly relevant for the description of the sampling design. Please also make sure it is consistent with your actual sampling design (i.e. not a random sample). In addition, please clarify how the qualitative analysis is done. As the reviewers recommend, you may consider analysing the relationships between the various variables, but restricting to a more descriptive analysis is also acceptable.

- We have removed the mention of the term ‘random’ when describing our sample and we have also updated the description of the qualitative analysis. Given that our data is shared openly and available to anyone looking to analyze our data further, we have restricted our discussion in our manuscript to focus on a descriptive analysis only.

[Note: HTML markup is below. Please do not edit.]

Reviewers' comments:

Reviewer's Responses to Questions

Comments to the Author

1. Is the manuscript technically sound, and do the data support the conclusions?

The manuscript must describe a technically sound piece of scientific research with data that supports the conclusions. Experiments must have been conducted rigorously, with appropriate controls, replication, and sample sizes. The conclusions must be drawn appropriately based on the data presented.

Reviewer #1: Yes

Reviewer #2: Partly

Reviewer #3: Yes

2. Has the statistical analysis been performed appropriately and rigorously?

Reviewer #1: Yes

Reviewer #2: N/A

Reviewer #3: I Don't Know

3. Have the authors made all data underlying the findings in their manuscript fully available?

The PLOS Data policy requires authors to make all data underlying the findings described in their manuscript fully available without restriction, with rare exception (please refer to the Data Availability Statement in the manuscript PDF file). The data should be provided as part of the manuscript or its supporting information, or deposited to a public repository. For example, in addition to summary statistics, the data points behind means, medians and variance measures should be available. If there are restrictions on publicly sharing data—e.g. participant privacy or use of data from a third party—those must be specified.

Reviewer #1: Yes

Reviewer #2: Yes

Reviewer #3: Yes

4. Is the manuscript presented in an intelligible fashion and written in standard English?

PLOS ONE does not copyedit accepted manuscripts, so the language in submitted articles must be clear, correct, and unambiguous. Any typographical or grammatical errors should be corrected at revision, so please note any specific errors here.

Reviewer #1: Yes

Reviewer #2: Yes

Reviewer #3: Yes

5. Review Comments to the Author

Please use the space provided to explain your answers to the questions above. You may also include additional comments for the author, including concerns about dual publication, research ethics, or publication ethics. (Please upload your review as an attachment if it exceeds 20,000 characters)

Reviewer #1: The paper reports descriptive results of a survey of researchers in one research area in medicine (Complementary, Alternative, and Integrative Medicine) about their understanding and practices related to open science. The study has been well-designed and executed and the presentation is good. The data and the questionnaire have been made available publicly. The findings are interesting and can inform future initiatives to promote and advance open science in this area. I can't see any major error in the paper (just one minor) that needs to be fixed or any area for major improvement. However, I have a few points that authors might consider or respond to.

- We kindly thank this reviewer for providing their feedback on our manuscript.

Twice it is mentioned that authors were randomly selected. I could not see in the research design where this random approach was deployed and the authors also say in the method that a convenience sample was used. This is contradictory. I believe the approach has been convenient sampling (all authors with an email published within a time frame in certain journals were invited and those who self-selected to participate, completed the survey). Therefore, the mention of 'random' should be removed from the paper.

- Thank you for catching this. We have removed the mention of ‘random’ from the paper.

A lot of demographic questions have been asked without making any use of them. I understand some of them provide contextual information, but generally, the best practice is not to ask a question if the information is not to be used. Not sure what value knowing about minorities or visibility adds here. Too late to change the questionnaire, but beyond providing context, have the authors explored or want to explore any demographic differences in practising or awareness of open science (e.g. are younger researchers more likely to practice OS)? The same can be done for different questions if authors want to explore their data any further (for instance did having any training in OS make a difference in practising it?) I understand if the authors decide to keep the results descriptive as is.

We had originally decided to keep the results descriptive because we have shared all of our data openly. That being said, based on this reviewer’s feedback, we have further added 6 crosstab files to OSF which provide stratifications by key demographic questions including gender, age, minority group, caregiver, region, and career stage.

I believe a bit more literature can provide a good context for some of the findings. The authors can compare some of the results with studies in other fields (e.g. many other studies have found fees as the main barrier to OA publishing). Moreover, I am wondering if more information about CAIM field might be helpful in understanding the results. The percentage of registering a protocol for instance is remarkably high (48%), maybe this is the norm or a requirement (by funders...). So any more information about this field might better explain the findings.

- We have added some additional comparative literature from other disciplines/fields which now reads “Comparative studies such as the survey of Molecular Biology Society of Japan members found that 76.6% (n = 478) respondents have published open access^35,41,42^. Additionally, 91.5% (n = 571) of respondents in the same survey reported wanting to publish open access^35^. Costs, however, remain a barrier to those wishing to publish open access, with some researchers having to use their personal funds towards APCs^35^. Ideally, OS practices such as open access publishing promote equity and reduce barriers that end-users and authors face. Tools such as [Sherpa Romeo](https://www.sherpa.ac.uk/romeo/) exist to help users understand individual journal policies regarding preprinting, post-printing, and open access publishing. Finding journals that lack APCs or imply charges should therefore be easier for readers and authors, which is especially important for those in lower-income countries.” However, studies looking at open science concerning CAIM are quite sparse. For example, we have provided additional context for the percentage of registering a protocol at the end of the discussion.

Reviewer #2: The authors mention that they make a qualitative analysis of the open-ended responses, but it is not clear from the text.

- We kindly thank this reviewer for providing their feedback on our manuscript. We have opted to keep the results of our study solely descriptive, as mentioned by the editor, and also because we have shared all of our data openly. With regards to the open-ended questions, all of the qualitative data was descriptively categorized. We have bolstered our methods section “Data Management, Analysis, and Reporting” to better describe this process.

In the methods it is said that the sample is random, but it is not.

- Thank you for catching this. We have removed the mention of ‘random’ when describing the sample.

The data on age, gender and professional group to which they belong is only visible in the raw data.

- We have represented this data in Table 3 of our manuscript.

Has any analysis of the reliability or consistency between some of the answers been done?

- We have not conducted an analysis concerning the reliability or consistency of our results. This has now been added as a limitation of our study. Our survey was also purpose built and future research could see to validate a tool to track these behaviors.

The paper does not make any statistical analysis of the data, it only gives percentages, it would have been interesting to see the crosstabs between e.g. gender, age, and the responses to the survey.

- We had originally decided to keep the results descriptive because we have shared all of our data openly. That being said, based on this reviewer’s feedback, we have further added 6 crosstab files to OSF which provide stratifications by key demographic questions including gender, age, minority group, caregiver, region, and career stage.

In the tables of the survey raw data, the number of responses is given with a decimal place in front, what does this figure mean?

- We have edited the raw data to properly indicate the percentage of participants who answered the question. Previously, this number was expressed as a decimal.

The work confirms what has already been seen in other disciplines but is somewhat weak because of the poor treatment of the data.

Reviewer #3: General:

- We kindly thank this reviewer for providing their feedback on our manuscript.

1. Authors should mention that a preprint version of this study is available on medRxiv 2023.10.24.23297458; doi: https://doi.org/10.1101/2023.10.24.23297458 .

- We have added the link to our preprint in the Methods Transparency Statement.

Abstract:

1. Please note that the study objectives presented in the abstract are not in alignment with those mentioned in other sections.

- We have revised our statement in the abstract “our objective was to assess practices and perceived barriers towards OS among CAIM researchers” so that it now closely aligns with the objective of our manuscript as well as the objectives mentioned in other areas of the manuscript.

2. Future research mentioned here - such as increasing CAIM researchers’ funding and educational resources to implement OS practices – is not aligned with what is mentioned in the conclusions of the manuscript, where you stated that - Future work can build upon our baseline study to monitor the implementation of open science interventions in the CAIM field. Please consider to align it with the conclusions presented at the end of the manuscript.

- We have changed the future work in the abstract to align with those mentioned in the conclusion.

Introduction:

1. As presented in your results, the authors consider that OA publication is very expensive and that more funding is needed to promote its adoption. So, in the literature review, it would be interesting to mention that there are several OA routes and that not all imply charges to readers or authors.

- We have noted preprinting, postprinting, as well as Sherpa Romeo where readers and authors can find detailed information regarding journal policies on these topics and APCs/costs. For example “Tools such as [Sherpa Romeo](https://www.sherpa.ac.uk/romeo/) exist to help users understand individual journal policies regarding preprinting, post-printing, and open access publishing. Finding journals that lack APCs or imply charges should therefore be easier for readers and authors, which is especially important for those in lower-income countries.”

2. When you mentioned - By limiting constraints on information access, the OS movement also supports equity - it would be interesting to not limit equity to the user's perspective. Equity also involves reducing or eliminating the barriers to the publishing system that authors face. Those from lower-income countries, for example, cannot pay for open access costs.

- We have noted the importance of lowering APCs and routes/tools such as preprinting and Sherpa Romeo to help authors and readers, especially those in lower-income countries. For example “Tools such as [Sherpa Romeo](https://www.sherpa.ac.uk/romeo/) exist to help users understand individual journal policies regarding preprinting, post-printing, and open access publishing. Finding journals that lack APCs or imply charges should therefore be easier for readers and authors, which is especially important for those in lower-income countries.” We have further added 6 crosstab files to the OSF which provide stratifications by key demographic questions including gender, age, minority group, caregiver, region, and career stage.

3. You mentioned - Knowing about CAIM researcher’s opinions of OS and the barriers they face may benefit the adoption of CAIM by the public, the further implementation of evidence-based practices in CAIM research, and the removal of stigmas regarding the unreliability and untrustworthiness of CAIM20. – Considering your study goals and results, you can not assume that your study will bring those benefits. Those are the benefits that OA can bring to health and medical research in general as often reported in the literature. As you mention - These surveys serve as the foundation for developing new interventions that more successfully implement open research (…). So, by exploring what are CAIM researchers’ practices and perceived barriers related to OS, you are only able to better understand the experiences of those authors and identify the need for further work to promote the adoption of OA practices in CAIM research.

- We have removed this wording and added an additional point that is more in line with the goals of this study. This section now reads “These surveys serve as the foundation for developing new interventions that more successfully implement open research, which can also be used to track the progression of OS using a longitudinal design. By gathering and understanding the practices and perceived barriers that CAIM researchers face concerning OS, we can then identify the best ways to increase OS adoption in the CAIM field.”

4. Please make sure that the goals listed here are in alignment with those in the abstract, discussion and conclusion – here you mention – Our goal was to explore (…), in the abstract and discussion – Our objective was to access, and in the conclusion you – In this study, we explored (…). Please be consistent

- We have changed the wording when referring to the goal of this study so that we are consistent throughout the manuscript. These sections now read “The goal of this study was to explore what are CAIM researchers’ practices and perceived barriers related to OS.”

Methods:

1. Study Design - Please make sure that the selected journals mentioned here – CAIM journals – are in alignment with those mentioned in the introduction section – journals indexed in Scopus.

- We have now added that the journals were indexed in Scopus.

2. Survey design - When you mentioned – The majority of the survey questions were multiple choice – please provide more detailed information: How many? What other types of questions were included? How questions were grouped? The questions were mandatory or optional? (…)

- We have updated this section of the manuscript to provide more detail on the survey questions. This now reads, “There were 32 multiple-choice questions and 2 open-ended questions. All questions were optional and could be skipped to proceed through the survey.”

3. In the Data Management, Analysis, and Reporting section, you mentioned – in respect to qualitative data (…) open-ended questions. Please make sure that the information mentioned here is aligned with the one mentioned in the Survey Design section, where no information regarding open-ended questions was mentioned. Please add more details on how qualitative data was analyzed and by whom.

- We have included mention of the open-ended questions in the survey design section and we have added more detail regarding how the qualitative data was analyzed in the data management, analysis and reporting section. This now reads “With respect to qualitative data, a thematic content analysis was conducted by two researchers who analyzed repeated ideas from the open-ended text-based responses. The researchers first collected, categorized, and grouped the responses into themes separately, followed by a discussion to obtain a final consensus of the themes and codes for reporting.”

Results:

1. In the Methods Section, you mentioned that the authors were selected at random, but here you say that all those who had an email available were contacted. You should clarify, otherwise it seems contradictory.

- Thank you for catching this. We have removed the term ‘random’ when describing the sample.

2. As the survey had open-ended options, please report how many respondents you had per each such question, how were they analyzed/coded and how many different categories you found for those answers.

- We have created two new tables (Table 4 and 5) to report the thematic analyses of the open-ended options. We have also edited the methods to better describe this analysis in more detail.

3. Please avoid using less, many, most or similar expressions without data like you did In the Using Reporting Guidelines section, where you mentioned - 1) many respondents report not (…) please specify n and %.

- We have now removed mention of these expressions where applicable in the results section.

Discussion:

1. You stated that – These results are consistent with previous studies – but then you only cite one study (ref. 32). Please consider enriching your literature review. Other studies besides Pardo Martínez C and Poveda A, like this recent one, https://onlinelibrary.wiley.com/doi/10.1111/gtc.13015, reporting the results of a questionnaire survey of MBSJ members about preprints and open access publishing, could be of interest for the discussion and likely could be included in the literature review.

- We have added some additional studies to the section mentioned above. We have also added some additional comparative literature to the discussion.

2. As mentioned before, when mentioning many or the majority (…) please add n and %.

- We have added the n and % where applicable throughout the manuscript.

Conclusion:

1. As mentioned before, when mentioning many or the majority (…) please add n and %. Also, make sure that the goals listed here are in alignment with those mentioned before – goals or objectives / explore or access.

- We have removed mention of many or the majority and we have made sure the goals mentioned in the conclusion are consistent with those mentioned in other areas of the manuscript.

2. As mentioned before future research mentioned here is not aligned with what is mentioned in the Abstract. We suggest you keep what you mention here - Future work can build upon our baseline study to monitor the implementation of open science interventions in the CAIM field - assuring a stronger final message, and not limiting OA to funding or education.

- We have ensured the future work mentioned in the conclusion is consistent with what is now mentioned in the abstract.

=====================================================================

Should your editorial office require any further edits following my most recently submitted submission, please do not hesitate to inform me and we will make these changes as soon as possible. Thank you for your consideration.

Yours sincerely,

Dr. Jeremy Y. Ng, MSc, PhD

Postdoctoral Fellow, Centre for Journalology, Ottawa Methods Centre, Ottawa Hospital Research Institute, Ottawa, Canada

Research Associate, Institute of General Practice and Interprofessional Care, University Hospital Tübingen, Tübingen, Germany & Robert Bosch Center for Integrative Medicine and Health, Bosch Health Campus, Stuttgart, Germany
